# CT Derived Hounsfield Unit: An Easy Way to Determine Osteoporosis and Radiation Related Fracture Risk in Irradiated Patients

**DOI:** 10.3389/fonc.2020.00742

**Published:** 2020-05-13

**Authors:** Gokhan Yaprak, Cengiz Gemici, Ozgur O. Seseogullari, Irem S. Karabag, Nilsu Cini

**Affiliations:** ^1^Department of Radiation Oncology, Kartal Dr. Lutfi Kirdar Education and Research Hospital, Istanbul, Turkey; ^2^Department of Radiation Oncology, Biruni University Medicana Hospital, Istanbul, Turkey; ^3^Department of Radiology, Ondokuz Mayis University, Samsun, Turkey

**Keywords:** radiation toxicity, bone, bone mineral density, hounsfield unit, osteoporosis

## Abstract

**Background:** We aimed to evaluate osteoporosis, bone mineral density, and fracture risk in irradiated patients by computerized tomography derived Hounsfield Units (HUs) calculated from radiation treatment planning system.

**Methods:** Fifty-seven patients operated for gastric adenocarcinoma who received adjuvant abdominal radiotherapy were included in the study group. Thirty-four patients who were not irradiated after surgery comprised the control group. HUs of T12, L1, L2 vertebral bodies were measured from the computerized tomographies imported to the treatment planning system for all the patients. While the measurements were obtained just after surgery and 1 year later after surgery in the control group, the same measurements were obtained just before irradiation and 1 year after radiotherapy in the study group. Percent change in HU values (Δ%HU) was determined for each group. Vertebral compression fractures, which are the consequence of radiation induced osteoporosis and bone toxicity were assessed during follow-up.

**Results:** There was no statistical significant difference in HU values measured for all the vertebrae between the study and the control group at the onset of the study. While HU values decreased significantly in the study group, there was no significant reduction in HU values in the control group after 1 year. significant correlation was found between Δ%HU and the radiation dose received by each vertebra. Insufficiency fractures (IFs) were observed only in the irradiated patients (4 out of 57 patients) with the cumulative incidence of 7%.

**Conclusions:** HU values are very valuable in determining bone mineral density and fracture risk. Radiation treatment planning system can be utilized to determine HU values. IFs are common after abdominal radiotherapy in patients with low vertebral HU values detected during radiation treatment planning. Radiation dose to the vertebral bones with low HU values should be limited below 20 Gy to prevent late radiation related bone toxicity.

## Background

Radiation induced osteoporosis and resulting insufficiency fractures (IFs) are very common. The incidence of IFs reported in the literature after abdominal or pelvic irradiations vary between 7 and 45% (1–8). In certain oncologic situations such as hormonal treatment of breast and prostate cancer, clinicians are familiar with treatment related osteoporosis and fracture risk (9–13). Dual-energy x-ray absorptiometry (DEXA) is ordered routinely and preventive measures against osteoporosis such as calcium, vitamin D, or biphosphonates are taken prophylactically during follow-up of the patients. However, there is no routine evaluation of bone health, and no awareness of radiation related bone toxicity and IFs among radiation oncologists. Although IFs are often encountered in the follow-up of the patients after abdominal or pelvic irradiations and observed nowadays with very high frequency after stereotactic irradiations, there is no consideration of DEXA or other alternative bone mineral density (BMD) measurements in the radiation oncology practice (1–8, 14–16). Due to unawareness of IFs by radiation oncologists, these IFs are often not recognized or sometimes can be considered as the bone metastases of the primary cancer resulting in malpractice and unnecessary examinations and psychological stress for patients.

We have recently demonstrated 9.6% vertebral fracture risk in patients who were treated with abdominal irradiation (8). We think that bone should be considered as an organ at risk for radiotherapy planning and BMD should be measured and followed regularly in these patients before and after irradiation. Radiation oncologists can determine the bone health of their patients and unrecognized IFs by using their most important tool: the radiation treatment planning system.

BMD is the measure of bone mineral and calcium density and it is determined by DEXA (17, 18). DEXA is currently considered to be the gold standard method for BMD quantification and has been shown to correlate with fracture risk and the efficacy of treatment (17, 18). Several studies have demonstrated that computerized tomography (CT) derived Hounsfield units (HUs), has a strong correlation with BMD provided by DEXA (19–22). HU values can provide reliable estimate for regional bone strength and BMD, and can be utilized to rule out osteoporosis with high accuracy (19–22). Furthermore, CT scan when compared with DEXA allows a more accurate identification of vertebral fractures (23). Diagnostic and radiation planning CT scans are ordered either to stage disease, plan radiation treatment or to follow-up of the patients routinely. We can utilize CT-derived HUs in these patients to determine and monitor BMD, and hence we can avoid extra cost and additional radiation exposure from DEXA measurement. Beside these advantages, sagittal vertebral views reconstructed easily from CT scans can be used to evaluate unrecognized and non-symptomatic bone fractures.

The aim of the present study is to demonstrate bone mineral density loss and undiagnosed vertebral fractures by measuring HUs and sagittal reconstruction of vertebrae from the CT scans imported to the treatment planning system for patients who are treated with abdominal radiotherapy.

## Methods

Fifty-seven consecutive patients operated for gastric adenocarcinoma who received adjuvant abdominal radiotherapy were included in the study group. Thirty-four consecutive patients with early stage disease who did not need adjuvant treatment after surgery comprised the control group.

In the irradiated patients, radiation was delivered with 6 or 15 MV photons by either conformal, intensity-modulated, or volumetric modulated arc treatment planning. The radiation dose prescribed was 46 Gy in 23 fractions with 2 Gy per day, or 45 Gy in 25 fractions with 1, 8 Gy per day, 5 days per week, for 5 weeks. All the patients received either bolus or infusional 5-fluorouracil, one cycle before, two cycles concomitant with, and one cycle after radiation treatment. Informed consent was obtained from all the patients and the study was approved by the local ethics committee of the hospital.

Planning and diagnostic tomographies were obtained using multidetector CT scanners (LightSpeed 16 slice or VCT 64 slice; GE Healthcare, Waukesha, WI) by either 3 or 5 mm slice thicknesses. Abdominal tomographies were imported from the radiology picture archiving and communication system to the Eclipse Treatment Planning System (TPS) (Varian Medical Systems, Palo Alto, CA). In the study group, the first tomography was already in the treatment planning system for radiation planning. The second one was obtained 1 year after radiotherapy and imported to the treatment planning system. In the control group abdominal tomographies obtained just after surgery and 1 year later were imported to the planning system. T12-L1-L2 vertebral bodies were contoured in order to determine mean radiation dose for each vertebral body in the irradiated patients by a radiation oncologist. After that, an experienced radiologist using TPS determined the mean HU values of the same vertebrae for all the patients. The mean HU values for both groups of patients were remeasured 1 year later.

HU values at the beginning of the study and those obtained 1 year later were calculated for each vertebral body at each group and the percent change in HUs (Δ%HU) was determined. Although the duration of HU measurements were planned at the beginning and 1 year later, we continued to follow the patients regularly for at least 5 years after treatment for oncological outcome. Vertebral compression fractures, if any developed, were determined from sagittal reconstruction of vertebrae from the computerized tomographies during the follow-ups.

### HU Measurement Technique

HUs were measured at the axial cross sections of the trabecular regions of T12, L1, L2 vertebral bodies. Each vertebral body was divided into three axial segments and HUs were calculated by placing rectangular region of interest (ROI) over an area of trabecular bone on the vertebral body. We tried to avoid the basivertebral venous plexus posteriorly and subchondral sclerotic bone. The measurements were detailed in Supplemental Figure 1. Mean HU values of three axial segments in each vertebra were averaged to determine the final HU value for each vertebral body. HU_pre_ represents HU value measured in the initial CT for both study and control groups while, HU_post_ defines HU value measured after 1 year.

### Statistical Analyses

Percent decrease in bone attenuation (Δ%HU) for each vertebra was calculated with the following equation:

$$\begin{array}{r} \Delta \text{\%HU}=\left(\text{H}{\text{U}}_{\text{post}}-\text{H}{\text{U}}_{\text{pre}}\right)/\text{H}{\text{U}}_{\text{pre}} \end{array}$$

Chi-square analyses were done to demonstrate the differences between age, gender, and tumor characteristics of the groups. Students *t*-test was used to determine the differences between pre and post HU-values in each group and between two groups, and for three radiation dose levels (<20 Gy, 20–40 Gy, and >40 Gy) in the study group. To directly assess the effect of radiation dose on the change in Δ%HU, Pearson's correlation analysis was performed on Δ%HU and radiation dose. We considered a *p*-value of < 0.05 as significant. The statistical analysis was performed using the Statistical Package for Social Sciences (SPSS) software 17.0.

## Results

The patient and treatment details are presented in Table 1. There was no statistically significant difference between the groups with respect to age, gender, surgical resection type, and tumor location. Mean HU values of T12, L1, and L2 vertebrae were measured in both groups. At the beginning of the study, there was no statistically significant difference in HU values measured for all the vertebrae between the study and the control groups. While the mean T12, L1, L2 vertebrae's HU values decreased significantly in the study group (*p* < 0.001 for each vertebra) after 1 year, no significant change in HU values were found in the control group (*p*: 0.09–0.08–0.24, respectively) during the same period. Table 2 summarizes the changes in HUs for the study and the control groups.

**Table 1:** Patient and treatment characteristics.

	**Study group** **Radiotherapy (+)**		**Control group** **Radiotherapy (–)**		***P***
	***N***	**%**	***N***	**%**	
**Gender (Male/Female)**	39/18		23/11		0.94
**Age**	59 (22-77)		59 (34–78)		0.68
**Surgical resection type**					0.85
Subtotal	29	61.7	18	38.3	
Total	28	63.6	16	36.4	
**Tumor location**					0.76
Cardia	15	60	10	40	
Corpus	15	65.2	8	34.8	
Pylorus	7	58.3	5	41.7	
Antrum	20	64.5	11	35.5	
**Tumor stage**					<0.001
T1	–	–	12	100.0	
T2	9	29	22	71	
T3	21	100.0	–	–	
T4	27	100.0	–	–	
**Nodal stage**					<0.001
N0	9	20.9	34	79.1	
N1	13	100.0	–	–	
N2	15	100.0	–	–	
N3	20	100.0	–	–	
**Radiotherapy technique**
3D-CRT^*^	38	66.7			
IMRT^**^	10	17.5			
VMAT^***^	9	15.8			
**Radiation dose**
45.0 Gy/1.8 Gy	38	66.6			
46.0 Gy/2 Gy	19	33.4			

* *3D-CRT, Three Dimensional Conformal Radiotherapy*;

** *IMRT, Intensity-modulated radiation therapy*;

*** *VMAT, Volumetric Modulated Arc Therapy*.

**Table 2:** Comparison of HUs in both groups themselves and in between the two groups for each vertebra.

	**Study group** **Radiotherapy (+)**	**Control group** **Radiotherapy (–)**	***P***
T12-HU_pre_	159.2 ± 42.1	158.1 ± 47.7	0.77
T12-HU_post_	108 ± 43.2	155.8 ± 51	<0.001
***P***	<0.001	0.09	
L1 – HU_pre_	158.7 ± 42.7	155.5 ± 47.3	0.73
L1 – HU_post_	109.9 ± 44.2	151.8 ± 49.5	<0.001
***P***	<0.001	0.08	
L2 – HU_pre_	156.7 ± 41	151.6 ± 47.6	0.59
L2 – HU_post_	111.7 ± 44.4	149.6 ± 50.5	0.001
***P***	<0.001	0.24	

**Figure 1:**
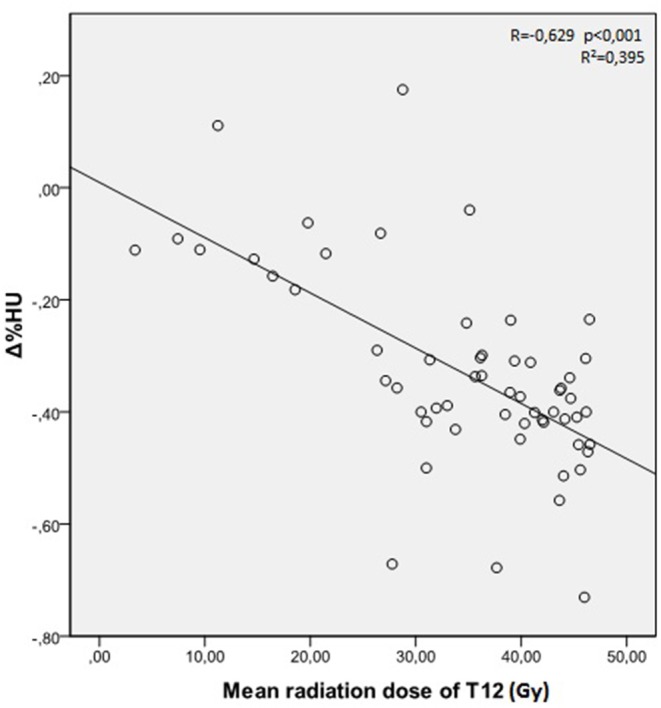
Correlation between Δ%HU and radiation dose received by T12 vertebra.

**Figure 2 F2:**
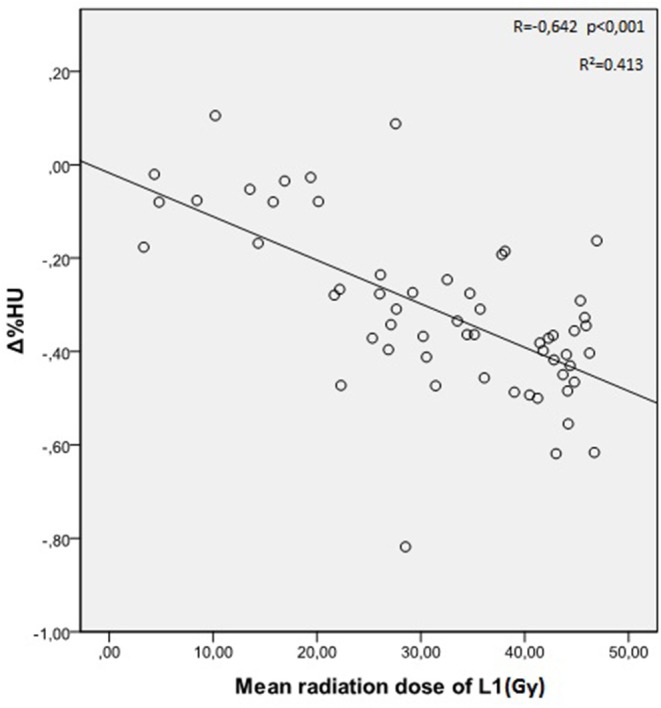
Correlation between Δ%HU and radiation dose received by L1 vertebra.

**Figure 3:**
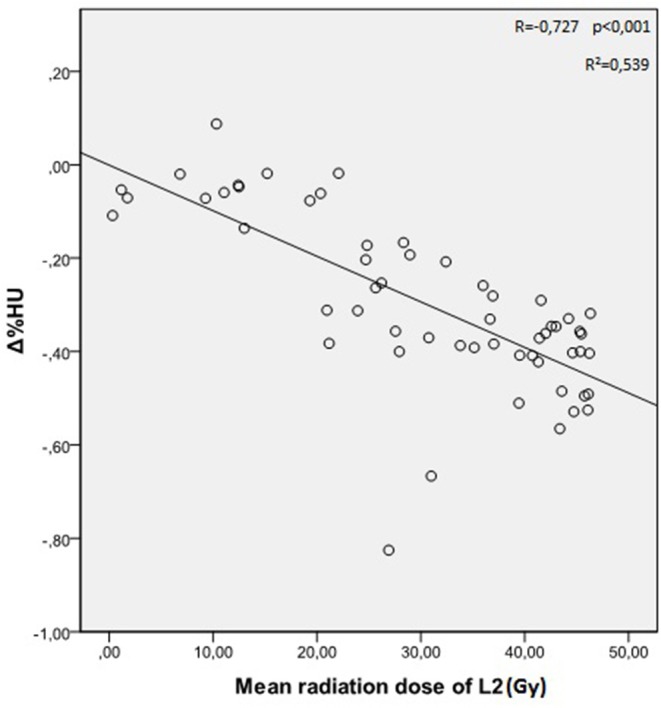
Correlation between Δ%HU and radiation dose received by L2 vertebra.

The mean radiation doses for T12, L1, and L2 vertebrae in the study group were 34.55 ± 11.1; 31.82 ± 12.4; 30.37 ± 13.6 Gy, respectively. A negative and significant correlation was found between Δ%HU and the radiation dose received by each vertebra. As the radiation dose increased, HUs decreased in each vertebra. This is summarized in Figures 1–3.[Fig F2]

The relation between the radiation dose received by vertebra and the HU change was summarized in Table 3. Although the decrease at the HU values were statistically significant at radiation dose levels above 20 Gy, HU values were not altered significantly in radiation doses below 20 Gy, except for L2 vertebrae.

**Table 3:** Change in HUs with respect to radiation dose groups.

	**<20 Gy**		**20 – 40 Gy**		**>40 Gy**	
	**Mean**	***P***	**Mean**	***P***	**Mean**	***P***
T12-HU_pre_	160.6 ± 48.6	0.09	158.4 ± 39.5	<0.001	159.7 ± 44.8	<0.001
T12-HU_post_	145.4 ± 44.1		109.1 ± 45.9		93.2 ± 30.9	
L1 – HU_pre_	159.2 ± 46.8	0.06	158.8 ± 44.6	<0.001	158.5 ± 40.4	<0.001
L1 – HU_post_	150.7 ± 50.7		108.3 ± 42.9		92.5 ± 29.3	
L2 – HU_pre_	156 ± 43	0.03	156.8 ± 38.6	<0.001	157 ± 44.7	<0.001
L2 – HU_post_	148.9 ± 44.6		108.8 ± 44.9		93.5 ± 29.2	

### Fractures

While no patient in the control group had fractures, 4 out of 57 patients (%7) in the irradiated group developed vertebral fractures throughout the course of study. While two of the vertebral fractures were observed in females, two of them were observed in males. Fractures were identified in the 16th, 18th, 20th, and 26th months after irradiation, with a median follow-up time of 24 months (range: 12–36 months). Fractures were observed in L1 vertebra in three patients, and in L2 vertebra in one patient. The mean radiation doses received by each fractured vertebra were as follows; 39, 28.5, 22.3, 31 Gy. Characteristics of patients with vertebral fractures are summarized in Supplemental Table 1 and Supplemental Figure 2.

## Discussion

Radiotherapy leads to osteoporosis by direct and indirect mechanisms and IFs develop frequently after pelvic or abdominal irradiations in clinical practice (1–8). IFs in vertebral column and pelvic region are common and represent an important late side effect of radiotherapy. However, radiation oncologists are not aware of radiation induced osteoporosis and bone toxicity. Due to unawareness of radiation induced bone toxicity, no special attention is given to prevent this important late complication. We, in our previous study (8), tried to emphasize the importance of this problem and reported a very high incidence of vertebral fractures after abdominal irradiations as in the patients irradiated for pelvic tumors (1–7).

The radiation oncologists during radiation treatment planning pay much attention to the radiation dose received by each organ at risk. Although grade III/IV late toxicity rates for these very closely followed complications are not above certain percentages, abdominal, or pelvic radiotherapy related bone toxicity and resulting fractures are indeed higher than the well-known grade III/IV late toxicities. While dose constraints are well-defined for organs known to be at risk and are determinant of final plan approval, this is not the case for bone tissue.

Even in the latest version of Common Terminology Criteria for Adverse Events (CTCAE) Version 5.0 there is no specific definition of bone toxicity resulting from irradiation (24). There is no toxicity evaluation criteria for bone in the radiation oncology practice. Neither in the LENT-SOMA late toxicity scoring tables, nor in the Quantitative Analyses of Normal Tissue Effects in the Clinic report (QUANTEC), bone has been defined as an organ at risk, and no dose and volume constraint has been defined for this tissue (25, 26). However, the previous studies and our recently published study demonstrated that osteoporosis and fracture risk should be taken into consideration seriously, and necessary precautions should be taken during the follow-up of irradiated patients to prevent radiation related fractures (1–8).

DEXA is the gold standard method for BMD quantification and routine screening for osteoporosis. Radiation oncologists do not order DEXA for their patients who receive pelvic or abdominal irradiation. They do not consult these patients with the endocrinologist for fracture risk assessment and presence of osteoporosis before any radiation treatment. However, as radiation oncologists, we can use our planning system in order to determine BMD and osteoporosis risk. These evaluations can be made easily via CT scans ordered either for staging of disease, or for radiation treatment planning and for follow-up of the patients. We can determine BMD decrease, osteoporosis, and unnoticed fractures by measuring HU values of bone from the CT scans of the patients imported to the planning system, and by constructing sagittal images of the irradiated bony areas.

It has been recently suggested that CT derived bone HU values can be used to identify patients with decreased BMD and osteoporosis (19–22, 27–30). Pickhardt et al. (22) clearly defined how bone HU values can be measured and used as an alternative to DEXA for establishing BMD and osteoporosis diagnoses. In these studies, while bone HU values below 100 are considered as indicative of osteoporosis, HU values between 100 and 160 are considered as indicative of osteopenia, and HU values above 160 demonstrate normal bone mineral density (19–22). Patients with HU values above 160 have normal bone density and thus no DEXA measurement and no concern for bone health is necessary. Patients who have HU values between 100 and 160 can be regarded as osteopenic, and they need early intervention for prevention of osteoporosis and fracture risk in the future. Patients with HU values below 100 should be considered as osteoporotic. Low HU values should alert the radiation oncologist for ulterior fracture risk in bony areas that will be exposed to radiation. These patients should be consulted with the endocrinologist before administering any radiation treatment. In studies comparing HUs with DEXA for bone mineral density and determination of osteoporosis, some patients falling into non-osteoporotic group by DEXA were detected with vertebral fractures during the determination of HUs from CT scans (22, 29–31).

In our study, we tried to find an easy way for radiation oncologists to diagnose osteoporosis and determine fracture risk. If we implement these measurements in to daily routine during radiation treatment planning, we can determine osteopenia, osteoporosis, and fracture risk in patients who will receive abdominal or pelvic radiotherapy and intervene early to prevent late radiation bone toxicity.

While there was no statistical significant difference in HU values of T12, L1, L2 vertebrae obtained at baseline and 1 year later in the control group, HU values decreased significantly in the irradiated patients. We found vertebral fractures only in the irradiated patients. Four patients out of 57 had vertebral fractures after a median follow-up of 24 months, with a 7% cumulative incidence rate of fractures. Although the follow-up time was short and most of the fractures were asymptomatic, we reported 9.6% fracture rate in our previous study, with a longer follow-up time, and most of these fractures were symptomatic (8). High bone fracture risk in these patient's merits attention and bone should be considered as an organ at risk. The reported time for development of fractures after radiotherapy ranged between 2 and 63 months (2, 14). Interestingly, fractures as grade IV late radiation toxicity are observed at higher incidence than other well-known late radiation toxicities such as radiation fibrosis, cystitis, proctitis, etc.

The decrease in HU was dose dependent. There was higher risk of decrease in bone HU values with higher radiation doses received by the vertebrae. However, we found that the decrease in HU values was not significant for the vertebral bodies which were exposed to radiation doses below 20 Gy.

In the literature, there are contradictory findings in terms of the radiation dose where BMD loss is observed. While a study demonstrated BMD loss in patients treated with 22.5 Gy pelvic irradiation, another study demonstrated no correlation between radiation dose and insufficiency fracture risk (32, 33). Wei et al. (33) reported that even 5 Gy vertebral radiation doses result with significant BMD reduction and IFs in patients who were treated with abdominal radiotherapy. They also recommended to limit radiation doses to vertebral bodies especially in patients with low HU values detected during radiation treatment planning. Thus, we should define a dose constraint for the vertebrae within the radiation field. Dose constraints may vary depending on the HU values measured during radiation treatment planning. We should try to decrease mean radiation doses for the vertebral bones within the radiation field, especially in the elderly, and already osteoporotic patients.

## Conclusions

Radiation induced osteoporosis and resulting insufficiency fractures (IFs) are very common. BMD decrease, osteoporosis, and unnoticed fractures can be detected by measuring HU values of bone from the CT scans of the patients imported to the planning system, and by reconstructing sagittal images of the irradiated bony areas. In patients with already low bone HU values detected during radiation planning, one can intervene early to restore bone health and prevent future radiation related fractures. In order to prevent the bone toxicity related to radiation, the radiation dose to the vertebral bones with already low HU values at the time of treatment planning should be limited below 20 Gy.

## Data Availability Statement

Materials described in the article, including all relevant raw data, can be freely available to any scientist wishing to use them for non-commercial purposes, without breaching participant confidentiality and can be obtained from the corresponding author.

## Ethics Statement

Our study has been performed in accordance with the Declaration of Helsinki. The study was approved by the ethics committee of the Dr. Lutfi Kirdar Kartal Education and Research Hospital (2017/514/109/2). Written informed consent has been obtained from all the participants in the study.

## Author Contributions

GY: guarantor of integrity of the entire study. GY and CG: study concepts and design. NC: literature research. GY, CG, IK, OS, and NC: clinical studies. GY: statistical analysis. OS: manuscript preparation. CG: manuscript editing. All authors read and approved the final manuscript.

## Conflict of Interest

The authors declare that the research was conducted in the absence of any commercial or financial relationships that could be construed as a potential conflict of interest.

## Abbreviations

IFs: Insufficiency fractures
DEXA: Dual energy x-ray absorptiometry
BMD: Bone mineral density
CT: Computerized tomography
HU: Hounsfield unit
Δ%HU: Percent change in HU
ROI: Region of interest
SPSS: Statistical package for the social sciences
CTCAE: Common terminology criteria for adverse event
QUANTEC: Quantitative analyses of normal tissue effects in the clinic.
