# Supplementary material for: CT Derived Hounsfield Unit: An Easy Way to Determine Osteoporosis and Radiation Related Fracture Risk in Irradiated Patients
Source: Front Oncol. 2020 May 13;10:742. doi: 10.3389/fonc.2020.00742 (PMC7237579; doi:10.3389/fonc.2020.00742)
Supplement: Supplementary file 1 [file Data_Sheet_1.docx]

**Supplemental Materials and Methods**

**Region of interest (ROI) delineation of the vertebrae for HU calculation**

We imported the computed tomography scans obtained either for treatment planning or for follow-up to Eclipse Treatment Planning System in order to evaluate the vertebral bone mineral density (BMD). The vertebral levels were determined on the sagittal views.

Each vertebral body was divided into three axial segments and HUs were calculated by placing manually the rectangular ROI over an area of trabecular bone on vertebral body. The size of the ROI was determined by drawing the largest rectangle possible by avoiding the basi-vertebral venous plexus posteriorly and subchondral sclerotic bone. Hounsfield unit values from 3 separate locations were measured: immediately inferior to the superior endplate, in the middle of the vertebral body, and superior to the inferior endplate. Eclipse treatment planning software (Varian, Palo Alto, CA) automatically calculates the average HU values in the ROI histograms for each image. The HU values from the three axial slices were averaged to give a mean HU value for each vertebra.


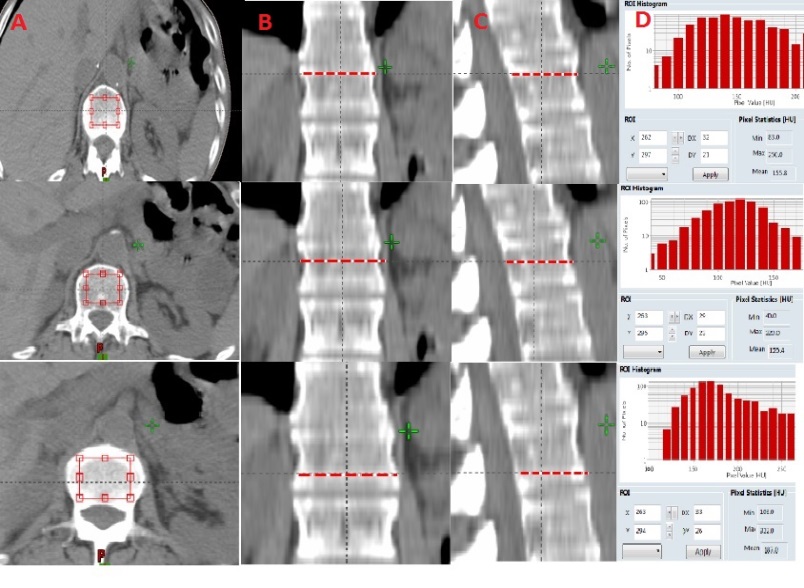


**Supplemental Figure 1:** Computerized tomography scans illustrating the technique for calculating vertebral BMD with HUs. Axial images, coronal images, sagital images demostrating placement of rectangular ROI (A–C). Minimum, maximum and mean HU values were obtained from ROI histogram tables constructed by the eclipse treatment planning system at each axial images (D).

**Vertebral Fracture Determineation**


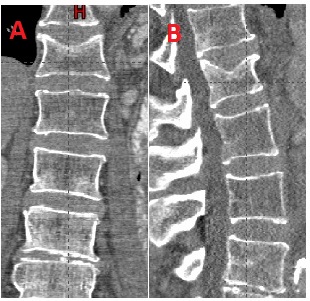


**Supplemental Figure 2.** Coronal (A) and Sagittal (B) CT views show a moderate insufficiency fracture (IF) in L1vertebrae

The CT scans that detected the vertebral IF were requested for follow-up of the patients without any complain for the fracture. None of the patients had evidence of metastatic disease when the vertebral fracture was detected.

**Supplemantal Table. 1**

| Patients | Age | Location | Initial HU | Mean Radiation Dose (Gy) | Latency Period (month) |
| --- | --- | --- | --- | --- | --- |
| 1 st | 67 | L2 | 126 | 31 | 16 |
| 2 nd | 63 | L1 | 119 | 39 | 18 |
| 3 rd | 73 | L1 | 154 | 28,52 | 20 |
| 4 th | 58 | L1 | 121 | 22,31 | 26 |
